# Characterization of glutathione S-transferase enzymes in *Dictyostelium discoideum* suggests a functional role for the GSTA2 isozyme in cell proliferation and development

**DOI:** 10.1371/journal.pone.0250704

**Published:** 2021-04-28

**Authors:** Mamatha Garige, Eric Walters

**Affiliations:** Department of Biochemistry and Molecular Biology, Howard University College of Medicine, Washington, DC, United States of America; Alexandria University, EGYPT

## Abstract

In this report, we extend our previous characterization of *Dictyostelium discoideum* glutathione S-transferase (DdGST) enzymes that are expressed in the eukaryotic model organism. Transcript profiling of *gstA1-gstA5* (alpha class) genes in vegetative, log phase cells identified *gstA2* and *gstA3* with highest expression (6–7.5-fold, respectively) when compared to other *gstA* transcripts. Marked reductions in all *gstA* transcripts occurred under starvation conditions, with *gstA2* and *gstA3* exhibiting the largest decreases (-96% and -86.6%, respectively). When compared to their pre-starvation levels, there was also a 60 percent reduction in total GST activity. Glutathione (GSH) pull-down assay and mass spectroscopy detected three isozymes (DdGSTA1, DdGSTA2 and DdGSTA3) that were predominantly expressed in vegetative cells. Biochemical and kinetic comparisons between rDdGSTA2 and rDdGSTA3 shows higher activity of rDdGSTA2 to the CDNB (1-chloro-2,4-dinitrobenzene) substrate. RNAi-mediated knockdown of endogenous DdGSTA2 caused a 60 percent reduction in proliferation, delayed development, and altered morphogenesis of fruiting bodies, whereas overexpression of rDdGSTA2 enzyme had no effect. These findings corroborate previous studies that implicate a role for phase II GST enzymes in cell proliferation, homeostasis, and development in eukaryotic cells.

## Introduction

Glutathione *S*-transferases (GSTs, EC 2.5.1.18) are phase II biotransformation enzymes that play important roles in cellular metabolism and detoxification. It is generally understood that GST enzymes function may complement phase I (cytochrome P450) biotransformation enzymes by mediating the conjugation of glutathione (GSH, tripeptide, γ-L- glutamyl-L-cysteinyl-glycine) to endogenous and exogenous substances, reducing toxicity and increasing solubility toward their excretion from cells [1]. GSTs catalysis converts GSH to an activated thiolate (GS^-^) nucleophile that is conjugated to electrophilic endobiotic and xenobiotic substrates (carcinogens, mutagens, reactive oxygen species, 2-oxoaldehydes, teratogens, clastogens) to reduce their interaction with cellular macromolecules [2, 3]. In addition to their antioxidant and detoxification properties, GSTs participate in the regulation of cell proliferation/apoptosis [4], metabolism of leukotrienes, prostaglandins, and steroids [5–7], indicative of both enzymatic and non-enzymatic function of GSTs in cellular homeostasis.

Mammalian GSTs constitute distinct and complex gene families that encode cytosolic, microsomal, and mitochondrial forms of alpha, sigma, kappa, mu, pi, theta, omega and zeta enzyme classes [8, 9]. GST classification is based on substrate selectivity, sequence similarity, isoelectric point, inhibitor sensitivity, and immunological properties. Each GST gene family includes subunits; the cytosolic enzymes are soluble and exist as homo or heterodimers of the same gene class. Across many species of prokaryotes and eukaryotes, GSTs are ubiquitously expressed within microsomes and/or the cytosol, with varying subunit specificities [10–12].

*Dictyostelium discoideum* is an eukaryotic soil amoeba that when starved, initiates cAMP signaling mechanisms to undergo chemotaxis, multicellular aggregation, and stage-specific morphogenesis that culminates in the formation of a spore-enriched fruiting body. Each developmental stage is controlled by complex signaling pathways. Possessing a haploid genome containing only six chromosomes [13], *D*. *discoideum* expresses many genes that are homologous to those of mammals, and thus has been employed as a model to study mechanisms of cell signaling, communication, chemotaxis, dynamics of social organization, and host-prey relationships [14, 15].

The coordinate action of phase I and phase II biotransformation during cellular development and differentiation in *D*. *discoideum* is poorly understood. Gonzalez-Kristeller and coworkers [16] reported that *D*. *discoideum* development and morphogenesis is stunted in CYP450 oxidoreductase null mutants. This finding emphasizes the importance of a phase I biotransformation in the developmental life cycle of *D*. *discoideum*, however the specific role(s) of GST enzymes in the *D*. *discoideum* life cycle remains unclear. Glutathione (GSH) depletion alters stage-specific programs of *D*. *discoideum* development [17, 18], noting the significance of GSH-dependent enzymes, such as GST, in *Dictyostelium* development and morphogenesis.

Gene sequence analysis of *D*. *discoideum* (strain AX4) reveals that five glutathione *S*-transferase genes encode for enzymes related to class alpha isozymes (*DdgstA1 –DdgstA5*). Although evidence of *DdgstA* transcription, activity and cellular localization of DdGSTs is reported in *D*. *discoideum* [19, 20], the role and contribution of individual isozymes in growth and development of the organism remains elusive. Our recent studies revealed that curcumin (a known modulator of GST enzyme activity) inhibited chemotaxis, proliferation, YakA signaling, and development in *D*. *discoideum*, and that these changes correlated with altered DdGST expression, activity, and localization [20]. The discovery that DGST4 isozyme may bind to the differentiation inducing factor (DIF-1) to regulate *Dictyostelium* development [21] indicates their importance and complexity amid enzymatic and non-enzymatic functions in eukaryotic cells [4, 22–24]. Thus, the *D*. *discoideum* model may be employed to elucidate the role of eukaryotic GSTs in programs of cell signaling and homeostasis.

In this report, we extend our previous characterization of GST enzymes in *D*. *discoideum* through biochemical and molecular genetic analyses of two DdGSTA isozymes. Gene expression profiling reveals that *D*. *discoideum* expresses five transcripts (*gstA1-5*) in vegetative amoeba, whose four isozymes presents altered expression is reduced under conditions of starvation. Mass spectrometric analysis of GSH-affinity protein isolates show that the DdGSTA2 and DdGSTA3 isozymes are predominant in vegetative cells. Biochemical and kinetic characterization of recombinant *D*. *discoideum* GST alpha (rDdGSTA2, rDdGSTA3) enzymes indicates that the rDdGSTA2 isozyme possesses higher activity toward the CDNB (1-chloro-2,4-dinitrobenzene) substrate, when compared to rDdGSTA3. RNAi-mediated knockdown of expressed *DdgstA2* mRNA and its protein enzyme correlated with reduced proliferation and delayed development, whereas overexpression of DdGSTA2 enzyme did not significantly alter growth and development. These findings provide the first evidence characterizing individual DdGST isozymes and corroborate previous findings for the importance of GSTs in *D*. *discoideum* homeostasis and development.

## Materials and methods

### *Dictyostelium discoideum* cell culture and development

*D*. *discoideum* (AX4 strain) cells were used for all experiments. AX4 cells were cultured in HL5 media containing 100 units/ml penicillin, and 100 μg/ml streptomycin at 22°C on a rotary shaker at 180 rpm. The conditions for AX4 propagation, cell culture, and development assays were conducted as described previously [20]. Cell counting and viability were assessed by hemocytometer and trypan blue staining.

### Real-time PCR

Comparative C_T_ method was used to calculate the relative gene expression of five *gstA* genes using *rnla* as internal control as described previously [20]. Amplified PCR products of the five *gstA* genes were sequenced to ensure the specificity of the primers.

### Primer sequences for qRT-PCR

**Table:** 

Gene	Forward (5’→3’)	Reverse (5’→3’)
*gstA1*	ATTGCCGCTAAACATGATTTCCT	CAGCGATTGTTTCATCAACGA
*gstA2*	ATTGTTACTGCAATTCACGCTG	GTTTTTGGAGTTTTTCTGGAACTGG
*gstA3*	CATGATTTCGTTGGTAAAACTCCAG	CAATATTGACAGCAGCGAGTG
*gstA4*	TTAGCCATTACCAAATGGGTTGG	CGAGAGCTCTTTCTTTGGATGA
*gstA5*	ACCAAAGAAGAAAGAGCAAGAGC	CCAAACAATGGTCCAAAACTTCC
*rnlA*	CGGATAAAAGGTACGCTAGGGATA	GTGCCGAACCACATAACAGATATG

### Glutathione-agarose affinity chromatography

Putative glutathione *S*-transferases from AX4 cells lysate were isolated with glutathione-agarose beads (G-4510, Sigma-Aldrich) using affinity chromatography protocols that were supplied by the manufacturer. Isolated protein species were resolved by SDS-PAGE analysis.

### Cloning and expression of recombinant *D*. *discoideum* (rDdGSTA2 and rDdGSTA3) GST enzymes

Total RNA from AX4 cells was isolated with Tri reagent (Sigma-Aldrich) and reverse transcribed into cDNA with M-MLV reverse transcriptase (Invitrogen). The coding sequence of the *gstA2* gene was amplified by PCR from cDNA, using specific forward and reverse primers designed to generate DNA flanked with *Xho*I (5’-TGGTGGCTCGAG*CTGGTGCCGCGCGGCAGC*ATGTCAAC- ATCATCAGTCCC-3’) and *Bam*HI (5’-GCGACGGGATCCTTATTAAAATTTAGTTTCTGGTCTTTCTTT-3’) restriction sites, respectively. The restriction enzyme sites are underlined in the primer sequences and the thrombin cleavage site in the forward primer is *italicized*. The amplified coding sequence of *gstA2* was cloned into a pET-15b expression plasmid (Novagen) at *Xho*I and *Bam*HI restriction sites to express an N-terminal 6X Histidine-tagged rGSTA2 enzyme.

The *gstA3* full-length cDNA coding region was cloned into the pET-15b expression vector as described above. The forward and reverse primers were designed to generate DNA with restriction sites *Nde*I (5’-TG- GTGGCATATG*CTGGTGCCGCGCGGCAGC*ATGACAA- AACCACAATTATC-3’) and *Bam*HI (5’GCGACGGGATCCTTATTAAATTTTTCTTTAGTAATTGGTC-3’), respectively. The restriction sites are underlined in the primer sequences and the thrombin cleavage site in the forward primer is *italicized*. The amplified PCR product was cloned into *Nde*I and *Bam*H1 of pET-15b to express the 6X Histidine-tagged rGSTA3 enzyme.

**Expression and purification of recombinant *D*. *discoideum* GSTA2 and GSTA3.** rGSTA2 and rGST3A proteins were expressed separately by transforming Single Step KRX E. coli competent cells (Promega) with cloned gstA2-pET-15b and gstA3-pET-15b plasmids. Recombinant protein expression was induced with 0.05% rhamnose/0.5mM IPTG (Isopropyl β-D-1-thiogalactopyranoside) and cells were lysed by sonication. The cell lysate was centrifuged at 12,000 rpm for 30 minutes at 4°C and supernatants containing rGSTA2 and rGSTA3 were loaded onto Ni-NTA columns (Novagen) that were pre-equilibrated with lysis/binding buffer. The column was washed twice with wash buffer (50 mM NaH_2_PO_4_, 300 mM NaCl, 5 mM β-mercaptoethanol, 50 mM imidazole, 5% glycerol, pH 8.0) to remove unbound proteins.

Protein concentrations of eluted, purified rGSTA2 and rGSTA3 was determined by absorbance at 280 nm using molar extinction coefficients of 19370 and 29340 M^-1^ cm^-1^, respectively. The purity of the enzymes was analyzed by Coomassie staining of SDS-PAGE gel, Western blotting [20] with a mouse anti-histidine antibody (Novagen) and mass spectrometric analysis (see below).

### Mass spectrophotometric analyses of rGSTA2 and rGSTA3 enzymes

In accord with the procedures of Ilinykh and coworkers [25], purified rGSTA2 and rGSTA3 were resolved on 10% SDS-PAGE [20] and stained with Simple-Blue Safe (Invitrogen). Gel pieces were excised, crushed, and were dehydrated and rehydrated (3X) with acetonitrile (Thermo Scientific) and 50 mM ammonium bicarbonate, respectively for 5 min. 10 mM DTT was used for reduction and alkylation of the cysteine residues at 60°C for 1 h followed by 50 mM iodoacetamide in the dark at room temperature for 30 min. In-gel digestion of proteins was done by adding trypsin at a final concentration 10 ng/μl and incubating at 37°C overnight. Eluted peptides were dried on a SpeedVac concentrator (ThermoFisher) and reconstituted in 0.1% TFA (trifluroacetic acid) and purified using C_18_Ziptips (Millipore). Liquid chromatography-mass spectrometry (LC-MS) analysis were performed on an LTQ Orbitrap XL mass spectrometer (ThermoFisher Scientific, Bremen, Germany) coupled to a Prominence Nano LC (Shimadzu, Columbia, MD) using the Xcalibur version 2.7.0 (Thermo Scientific). Forty (40μl) of reconstituted tryptic peptides in 0.1% formic acid were loaded on a C_18_-packed analytical column (25 cm × 150 μm, 5 μm, 200 Å, Michrom Bioresources, Auburn, CA) and separated with a linear gradient of 6–55 min, 2–40% B, 55–62 min, 40–80% B, 62–70 min, 80% B (v/v) at the flow rate of 600 nL/min. The mass spectrometer performed a full MS scan (*m/z* 300–2000) at a resolution of 30000. The three most intense ions were selected for fragmentation using collision-induced dissociation (CID) in the LTQ (normalized collision energy of 35); proteins were identified from the mass spectra results with Proteome Discoverer software (v 1.2).

### Measurement of GST enzyme activity

GST activity assays were conducted with 1-chloro-2,4- dinitrobenzene (CDNB) according to previous protocols [20, 26]; with the change in absorbance at 340 nm was measured at 25°C for 6 mins at 30s intervals in a microplate reader. A change in absorbance per min was converted into micromoles of substrate conjugated/min/mg of protein by adjusting the molar extinction coefficient of CDNB ε340 9.6 mM^-1^ cm^-1^ to 5.3 mM^-1^ (path length 0.552 cm). In our hands, the presence or absence of the N-terminal 6xHis tag of rGSTA2 and rGSTA3 did not alter full activity of the enzymes.

Affinity-purified rDdGSTA2 and rDdGSTA3 protein concentrations were determined by A260/280 spectrophotometry, and 5 ug of protein was used in each reaction. Assays were performed at 25°C at pH range from 3–9 with 1 mM GSH and 1mM CDNB as substrates in a 200 μl reaction volume. For pH 3.0–7.0, 100 mM citrate-phosphate buffer; pH 6.0–8.0, 100 mM potassium phosphate buffer and pH 8.0–9.0, 100 mM Tris-HCl buffer was used. The maximal enzyme activity is plotted as 100% relative activity.

GST kinetic assay for calculating K_m_ and V_max_ of GSH for rGSTA2 and rGSTA3 enzymes was assayed with CDNB at a saturating concentration and different concentrations of GSH. For calculation of K_m_ and V_max_ of CDNB, GSH was used at saturating concentration and CDNB at different concentrations. K_m_ and V_max_ were calculated by non-linear regression analysis of hyperbolic plots (*V* vs. *S*) using the Michaelis-Menten equation. GraphPad Prism 5 software was used for the analysis.

$$V=\frac{V_{max}\;x\;\left[S\right]}{\left[S\right]\;+\;K_{m}}$$

### Production of polyclonal anti-rDdGSTA2 antibodies

Removal of the 6X Histidine tag from the rGSTA2 purified enzyme was performed (via thrombin cleavage), and validation of rGSTA2 purity was confirmed by SDS-PAGE/ Coomassie staining. The purified rGST2A protein was suspended at a concentration of 1mg/ml and a rabbit anti-rGSTA2 polyclonal antisera was produced by Pocono Rabbit Farm and Laboratory Company (Candensis, PA). Western blot analysis of rGSTA2 and cell lysates was used to confirm the specificity of the antisera (see below).

### Construction of plasmid vectors for RNAi knockdown and overexpression of the DdGSTA2 isozyme

#### RNAi-*gstA2* Knockdown

RNAi-mediated knockdown of *gstA2* expression was accomplished by exogenous expression of a stem-loop RNA structure to target the *gstA2* mRNA transcript. To generate a stem-loop RNA, two fragments of the *gstA2* cloned sequence, which differed by 100 bp, were PCR amplified using a series of primers. Fragment 1 (sense strand) of 300 bp length was amplified from +14 of *gstA2* cDNA using two primers: Primer1 (forward, 5’-TGGTAGATCTCAAGTTTAACTTATTTCCAAGGTC-3’) was designed to amplify cDNA fragment with *Bgl*II restriction site (underlined); Primer2 (reverse, 5’-GCGAACTAGTGATATCCAAATGAACCAAAGTATTTTTCATAAAG-3’) was designed to generate adjacent *EcoRV*-*Spe1* restriction sites (underlined). Fragment 1 was cloned into *Bgl*II and *Spe1* arms of the doxycycline inducible vector pDM310 [27] to generate a plasmid containing the linear sequence of Fragment 1 (pDM310-Frag1).

Synthesis of Fragment 2 involved the utilized Primer 3 (forward, 5’-TGGTACTAGTCAAGTTTAACTTATTTCCAAGGTC-3’) and Primer 4 (reverse, 5’-GCGAGATATCACTGGATAAGGCTGCTTC-3’) to generate a 400bp fragment with *Spe*1 (underlined) and *EcoR*V (underlined) restriction sites, respectively. Fragment 2 was ligated in reverse orientation to pDM310Frag1 (using compatible sites of *EcoR*V and *Spe*1) to generate the pDM310-RNAi-*gstA2* (RNAi-*gstA2*) plasmid that would express a stem-loop silencer.

#### rGSTA2 overexpression (OE)

To overexpress the rGSTA2 enzyme, the complete coding region of *gstA2* was amplified by PCR from a cloned cDNA using specific forward (5’- TGGTAGATCTATGTCAACATCATCAGTCCC- 3’) and reverse primers (5’-GCGAACTAGTTTAAAATTTAGTTTCTGGTCTTTCTTT-3’) that incorporated *Bgl*II and *Spe*I restriction sites, respectively. The amplified product was digested and ligated to pDM310 at *Bgl*II and *Spe*I sites to generate the pDM310-*gstA2*-OE (*gstA2-*OE) plasmid.

### Transformation and expression of pDM310-RNAi-*gstA2* and pDM310-*gstA2*-OE in *D*. *discoideum*

Transformation of *D*. *discoideum* AX cells was performed in accord with previously established protocols [28–30]. Exponentially growing cells at a density of 1–2 x 10^6^ cells/ml were washed and resuspended in ice cold H-50 electroporation buffer (20mM Hepes pH 7.0, 50mM KCl, 10mM NaCl, 1mM Mg_2_SO_4_, 5 mMNaHCO_3_, 1mM Na_2_HPO_4_) at a concentration of 1X10^8^ cells/ml. To a cold 0.1 cm electroporation cuvette, 100 μl of cell suspension and 20μg of pDM310, pDM310-RNAi-*gstA2*, or pDM310-*gstA2*-OE were added and electroporated at 0.85KV/cm twice with 5 second intervals. CaCl_2_ and MgCl_2_ was added to the electroporated cells at a final concentration of 1 mM each and incubated at room temperature for 15 minutes to allow the cells to heal and cells were cultured in HL5 medium overnight. The next day, the culture medium was replaced with selection medium (HL5 with 10 μg/ml G418). Clonal selection was made by diluting the cells and plating them in microtiter plates.

RNAi-*gstA2* knock-down cells and *gstA2*-OE cells were cultured and maintained in HL5 media supplemented with 10 μg/ml G418, 100units/ml penicillin and 100 μg/ml streptomycin. To induce the knockdown *gstA2* transcripts or *gstA*2 overexpression, cells were exposed to doxycycline (10μg/ml) for a minimum of 12-24h. Proliferation of cells in the presence or absence doxycycline were quantified according to previous methods [20].

Semi-quantitative PCR was used to calculate the relative gene expression of *gstA2* to validate transcriptional knock-down and overexpression (OE) in transfected cell lines, using *rnla* as internal control [20]. An initial denaturation was performed at 94°C for 4 mins, followed by 18 cycles each containing of three steps: 30 seconds at 94°C, 30 seconds at 50°C, 40 seconds at 72°C. The normalized values are expressed as percent decrease or increase in expression as compared to controls.

Western blotting of lysates from RNAi-*gstA2* and *gstA2*-OE cells was done by lysis in RIPA (radio immunoprecipitation assay) buffer containing protease inhibitors (Sigma-Aldrich), followed by electrophoresis and immunoblotting with rabbit anti-rDdGSTA2 for 1hour at room temperature (1:2500 dilution). After rinsing, the blot was incubated with anti-rabbit HRP (horse radish peroxidase) linked secondary antibody for 1 hour. Chemiluminescent signals were captured using VersaDoc 3000 Imaging System (Bio-Rad) and quantified using ImageJ.

For developmental studies, cells were grown in HL5 media (expressing the RNAi-*gstA2* and *gstA2*-OE constructs) and then starved by washing three times in 1X KK2 buffer. Concentrated cells were spotted as 5 μl droplets on non-nutrient agar plates with or without 50 μg/ml doxycycline.

### Statistical analysis

All statistical analyses were performed using GraphPad Prism 5.0 software. Results are expressed as mean values ± standard deviation (SD). The student’s t-test was used for all analyses with a significance value of p ≤ 0.05.

## Results

### Characterization of *gstA* transcription and GST Enzyme activity in *D*. *discoideum*

**Fig 1:**
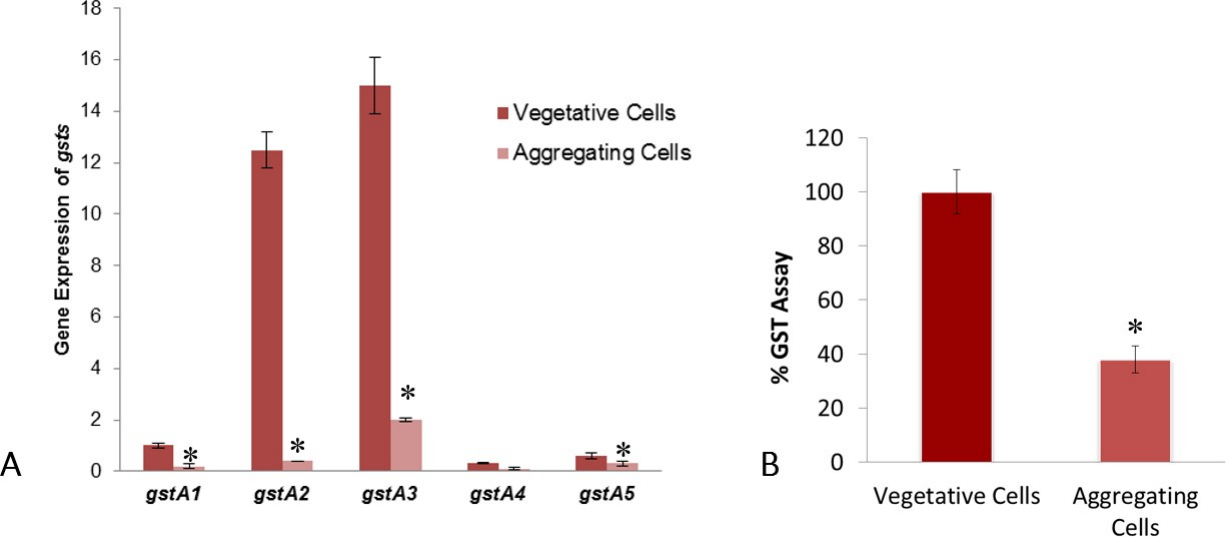
Transcription and Functional Profile of *D*. *discoideum* GST isozymes. (A) Quantitative PCR analysis of the gstA1-5 in vegetative and starvation-induced aggregating cells. (B) GSTs activity (CDNB conjugation) in vegetative and aggregating cells. Histograms represent at least three experiments (n = 3) performed in triplicate (mean ±SD, p<0.05).

The transcriptional profiles of five *D*. *discoideum* glutathione S-transferases alpha genes (*gstA1-A5*) were analyzed in axenic (vegetative log phase) and 6hr starved (aggregation-induced) cells by quantitative PCR. In vegetative cells, all transcripts of *gstA* genes were present; *gstA2* and *gstA3* displayed the highest expression as compared to other genes, while *gstA4* and *gstA5* were least expressed. When the cells were starved to induced aggregation, a decrease of *gstA1-5* expression was observed, with marked reduction of *gstA2* and *gstA3* (85% and 90%, respectively) when compared to their levels in axenically growing cells (Fig 1A).

Amino acid sequences of DdGSTA1-A5 proteins indicate that four of the five isozymes possess glutathione (GSH) binding motifs that are important for S-glutathionylation and substrate binding (Table 1). The collective decrease in *gstA1-A5* transcripts in starved cells correlated with reduced GST enzymatic activity (GSH-CDNB conjugation), as a 60% reduction of GST activity was found in starved cells as compared to vegetative cells (Fig 1B).

**Table 1:** Amino acid sequence analysis of *D*. *discoideum* GST Isozymes.

Name/Accession No.	Length (AAs)	N-terminal domain positions (AAs)	“G” site GSH binding (AAs)	C-terminal domain positions (AAs)	“H” site substrate binding (AAs)
GSTA1 Q55FF3	202	4–81	10 (Tyr)*, 51–52, 65–66	83–200	98, 101–102, 104–105, 155, 158
GSTA2 Q556G3	198	5–81	11(Tyr)*, 52–53, 65–66	83–198	98, 99, 102–103, 149, 152
GSTA3 Q54VI4	204	2–79	8(Tyr)*, 49–50, 63–64	81–202	96, 99–100, 103–104, 154, 157
GSTA4 Q86AU1	225	8–85	8(Ser)*, 14(Ser)*, 55–56, 69–70	87–207	102, 106–107, 110–111, 160, 163
GSTA5 Q54QV7	198	2–78	8*, 49–50, 62–63	80–198	95, 98–99, 102–103, 155, 158

Characteristic amino acid (AA) sequence and structural domains show *D*. *discoideum* GSTs contain an N-terminal thioredoxin-fold domain and a C-terminal alpha helical domain. Features indicate that putative active sites are formed between the two domains to form the co-substrate binding site (H-site). Asterisks (*) indicate the amino acid location of tyrosine (Tyr; GSTA1-A3, GSTA5) or serine (Ser; GSTA4) residues associated with binding to glutathione (GSH).

**Fig 2:**
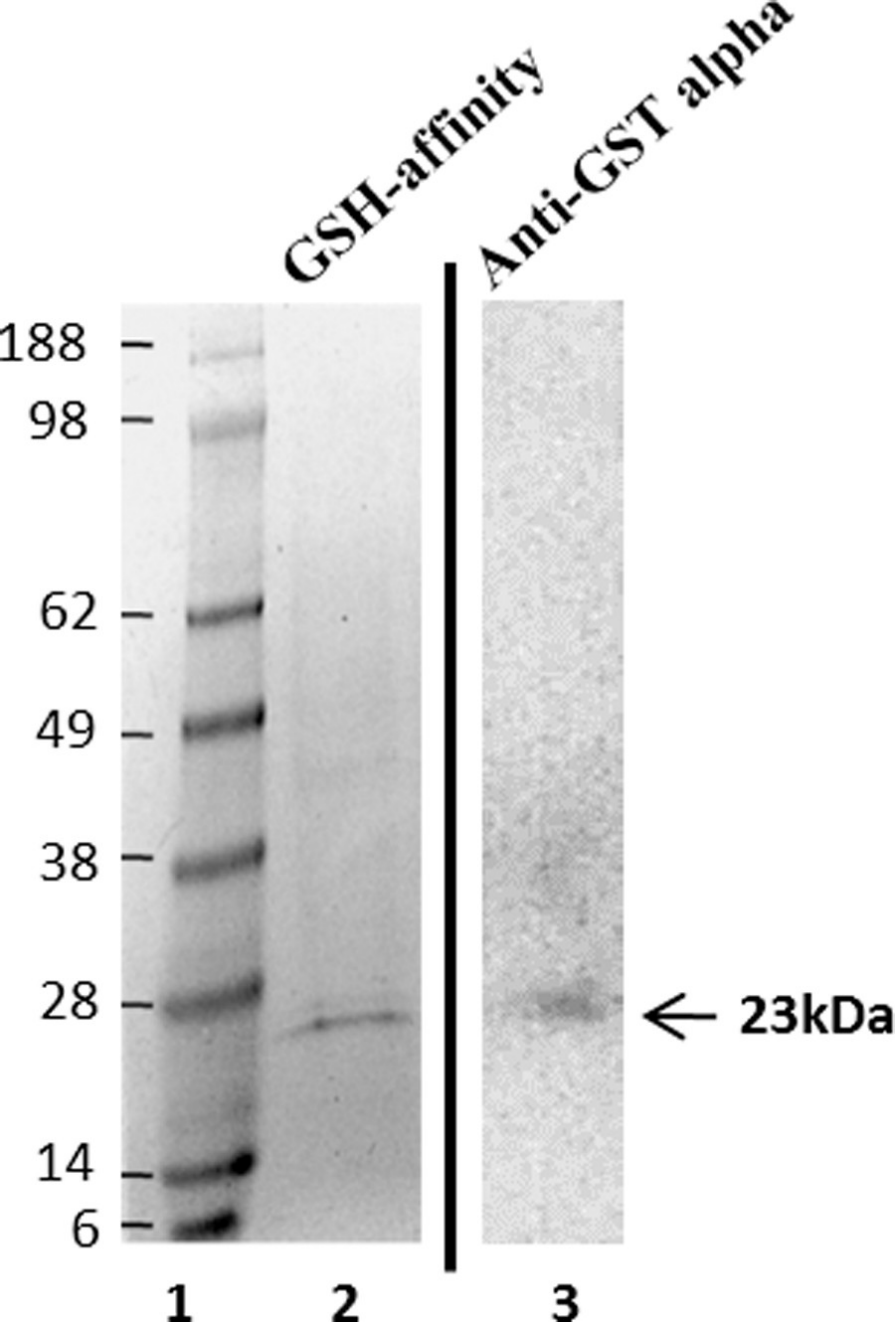
Presence of GST isozymes in *Dictyostelium* whole cell extracts. Lane 1: Protein marker. Lane 2: Coomassie staining of GSH-affinity isolated proteins from *D*. *discoideum* cell lysates. Lane 3: anti-GST alpha immunoblotting of GSH isolate with mouse anti-GST alpha polyclonal antibody (Novacastra Labs).

The identification of individual GST enzymes in log phase axenic cells was conducted by subjecting whole cell lysates to GSH-sepharose affinity chromatography. SDS-PAGE analysis of the isolated proteins and Western blot analysis with mouse anti-GST alpha polyclonal antibody displayed a distinct, approximately 23 kDa species (Fig 2) that corresponded to the amino acid molecular mass predictions for putative *D*. *discoideum* GST enzymes (DdGSTs). Mass spectrometric analysis of the isolated species identified three predominant GST isozymes, consisting of DdGSTA1 (23 kDa), DdGSTA2 (22.5 kDa) and DdGSTA3 (23.6 kDa) (Table 2). Notwithstanding DdGSTA1, the GST isozyme expression (of DdGSTA2 and DdGSTA3) in vegetative cells corresponded with the transcription profiles previously identified in vegetative cells (Fig 1A).

**Table 2:** Mass spectrophotometric analysis of GSH-affinity proteins from cell lysates.

Accession No.	MW [kDa]	Score	Description
Q556G3	23.0	198.01	Putative Glutathione S-Transferase alpha-3, OS = *Dictyostelium discoideum*, GN = gsta3
Q54VI4	23.6	49.82	Putative Glutathione S-Transferase alpha-1, OS = *Dictyostelium discoideum*, GN = gsta1
Q55FF3	22.5	34.76	Putative Glutathione S-Transferase alpha-2, OS = *Dictyostelium discoideum*, GN = gsta2

### Characterization and kinetic parameters of rDdGSTA2 and rDdGSTA3

**Fig 3:**
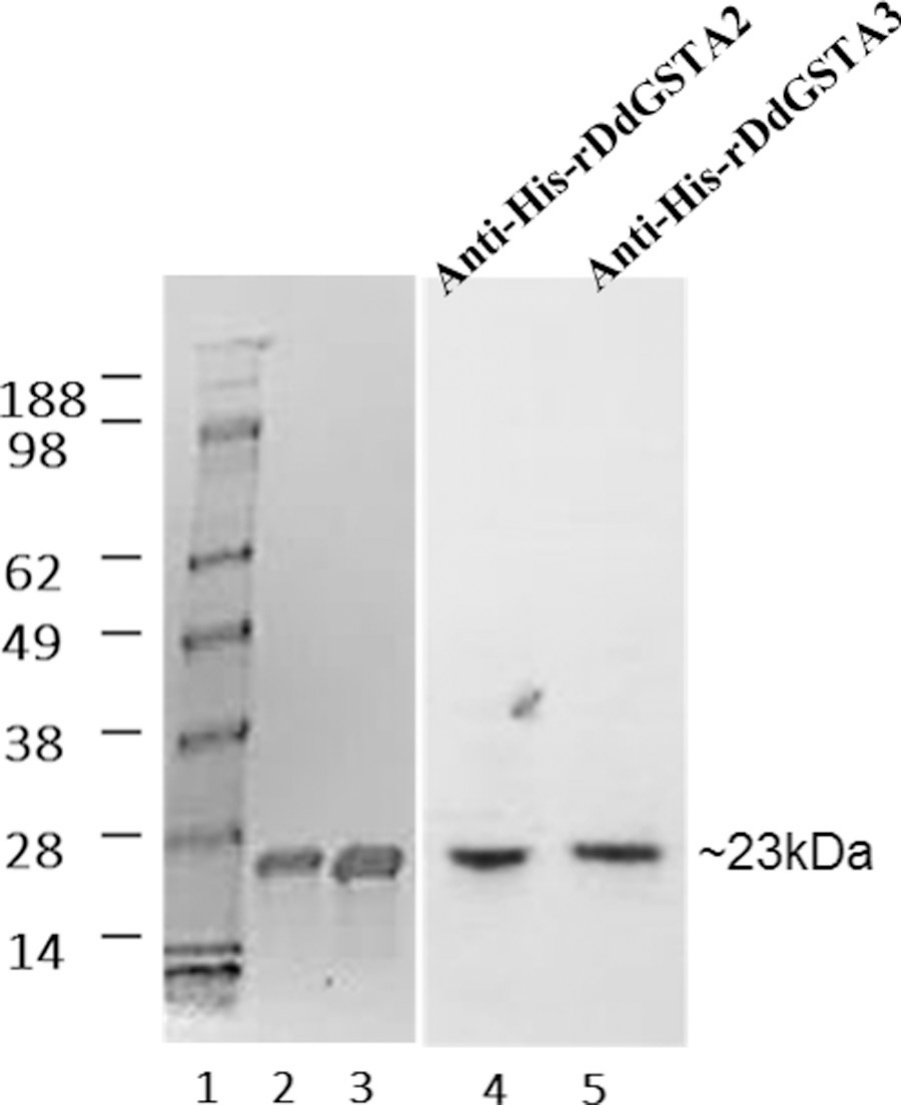
Expression and characterization of recombinant *D*. *discoideum* GST isozymes. Lane 1: Protein marker; Lanes 2–3: Coomassie staining of purified (6x His-tagged) rDdGSTA2 and rDdGSTA3 proteins; Lanes 4 and 5: Western blotting (anti-histidine monoclonal) of purified rDdGSTA2 and rDdGSTA3 enzymes, respectively.

The marked changes in *gstA2 and gstA3* transcription suggested that they may participate in growth and development of the organism. As a first step, we examined the biochemical and enzyme kinetic properties of rDdGSTA2 and rDdGSTA3 isozymes by generating recombinant proteins. SDS-PAGE and Western blot analysis of isolated Histidine-tagged rDdGSTA2 and rDdGSTA3 isozymes revealed independent, purified, and homogeneous species (Fig 3), each with a molecular mass of approximately 23 kDa. The identity, molecular mass, scores and the calculated isoelectric points (pI) of the two DdGST isozymes by mass spectrometry are listed in Table 3.

**Table 3:** Mass Spec analysis of purified rDdGSTA2 and rDdGSTA3.

Protein	Accession No.	Score	Amino Acids	MW [kDa]
rDdGSTA2	Q556G3	457.8	198	22.5
rDdGSTA3	Q54VI4	340.4	204	23.6

**Fig 4:**
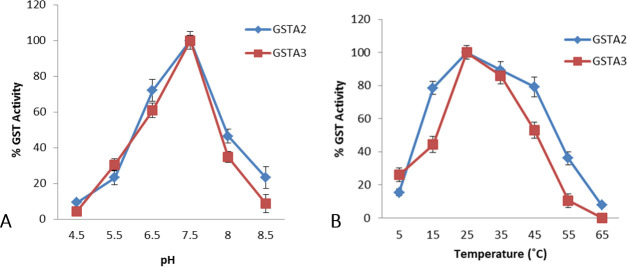
Optimal pH (A) and temperature (B) of rDdGSTA2 and rDdGSTA3 of *D*. *discoideum*. A standard GST assay (CDNB conjugation) indicated the optimal pH and temperature for maximal activity of rDdGSTA2 and rDdGSTA3 were 7.5 and 25°C respectively.

Enzymatic activity of the recombinant DdGSTA isozymes was analyzed in the presence of 1 mM GSH and 1 mM CDNB (1-Chloro-2,4-Dinitrobenzene) as substrate. Both recombinant isozymes exhibited similar profiles of activity regarding pH and temperature. The effect of pH on the purified rDdGSTA2 and rDdGSTA3 isozymes showed a positive correlation between increased pH and enzyme activity. For rDdGSTA2 and rDdGSTA3 isozymes, optimal activity was reached at pH 7.5 (Fig 4A), and the optimal temperature for maximal activity for both the isozymes was 25°C (Fig 4B).

**Fig 5:**
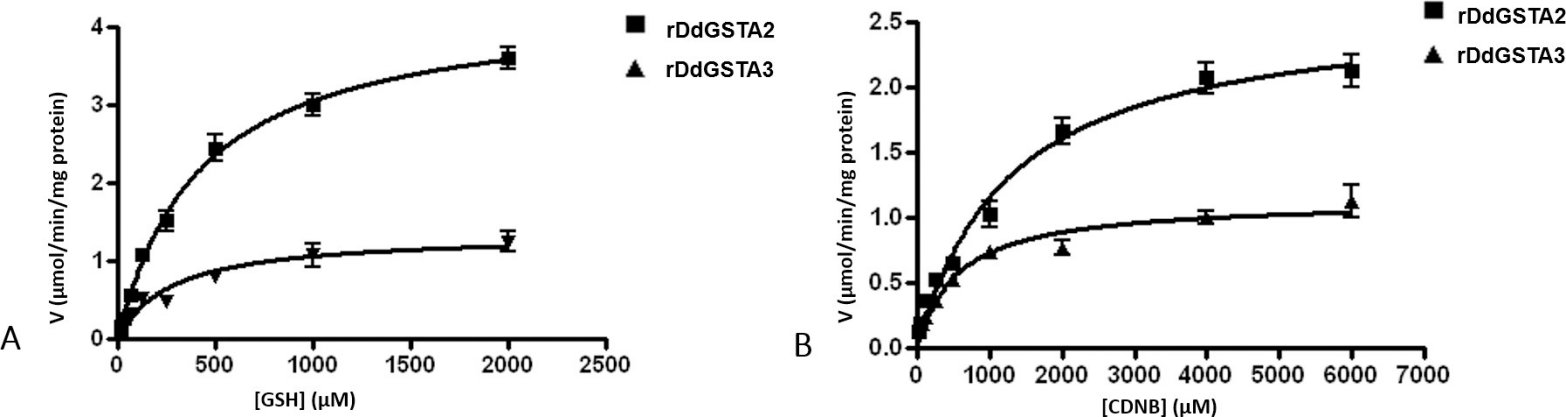
Kinetics of rDdGSTA2 and rDdGSTA3 isozymes. (A) Substrate versus velocity curves for calculation of K_m_ and V_max_ of GSH, for rDdGSTA2 and rDdGSTA3. GST kinetic assay was conducted in presence of saturating concentration of CDNB and different glutathione concentrations (0–2000 μM). (B) Substrate versus velocity curves for calculation of K_m_ and V_max_ of CDNB, for rDdGSTA2 and rDdGSTA3. GST kinetic assay was conducted in presence of saturating concentration of glutathione and different CDNB concentrations (0–6000 μM). Data are representative of three separate experiments (n = 3) and are given as mean ± SD (p≤0.05).

Steady state kinetics of rDdGSTA2 and rDdGSTA3 isozymes was studied at pH 7.5 and 25°C. The kinetic parameters (K_m_ and V_max_) of GSH, for rDdGSTA2 and rDdGSTA3 were calculated by maintaining CDNB at saturating concentration, while varying the concentrations of GSH (Fig 5A). The specific activity of rDdGSTA2 and rDdGSTA3 towards GSH was 4.3 and 1.36 μmol/min/mg protein, respectively. K_m_ and V_max_ for CDNB was calculated with a saturating concentration of GSH and different concentrations of CDNB (Fig 5B). The specific activity of rDdGSTA2 and rDdGSTA3 towards CDNB was 2.78 and 1.04 μmol/min/mg protein, respectively. K_m_ and V_max_ values for the isozymes are summarized in Table 4.

**Table 4:** Calculated K_m_ and V_max_ values for rDdGSTA2 and rDdGSTA3 Isozymes for GSH and CDNB.

Protein	K_m_ for GSH (μM)	V_max_ for GSH (μmoles/min/mg)	K_m_ for CDNB (μM)	V_max_ for CDNB (μmoles/min/mg)
rDdGSTA2	418.5 ± 42.33	4.334 ± 0.1615	1287 ± 200	2.643 ± 0.1444
rDdGSTA3	274.9 ± 69.76	1.356 ± 0.1130	561.9 ± 98.09	1.139 ± 0.0562

### Transcription of *gstA2* during multi-stage development in *D*. *discoideum*

**Fig 6:**
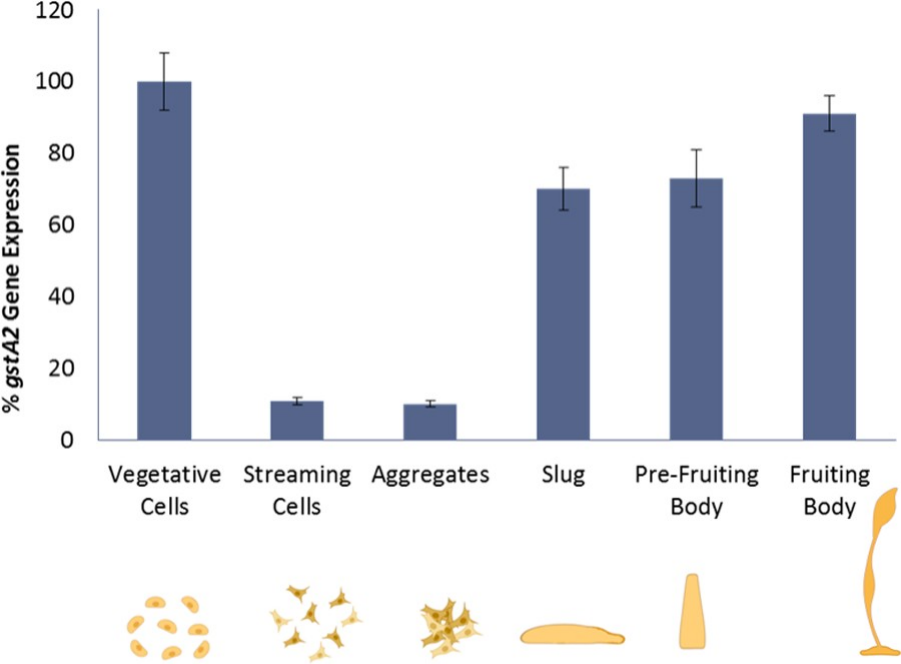
Transcriptional analysis of *gstA2* at different developmental stages of *D*. *discoideum*. In comparison to vegetative cells, *gstA2* expression is lower at initial stages of development and increases during stages of differentiation and morphogenesis. Histograms represent at least three experiments (n = 3) performed in triplicate (mean ±SD, p<0.05).

Sequencing of the AX4 genome indicates the presence a full-length duplication of the *gstA2* gene [31]. This, consonant with the comparatively elevated *gstA2* transcription in vegetative cells and the higher specific activity of rDdGSTA2 toward GSH, suggests its importance in *D*. *discoideum* homeostasis and development. Previous studies using microarrays indicated that *gstA* expression is elevated during later stages of development, suggesting that GSTs may be important during prespore development [32]. Our analysis of *gstA2* expression at various developmental stages revealed that *gstA2* dramatically decreases at the onset of streaming and aggregation, rebounds and increases as the morphogenesis proceeds toward culmination, slug, and fruiting body formation (Fig 6).

### RNAi-mediated knockdown and overexpression of the *gstA2* gene

**Fig 7:**
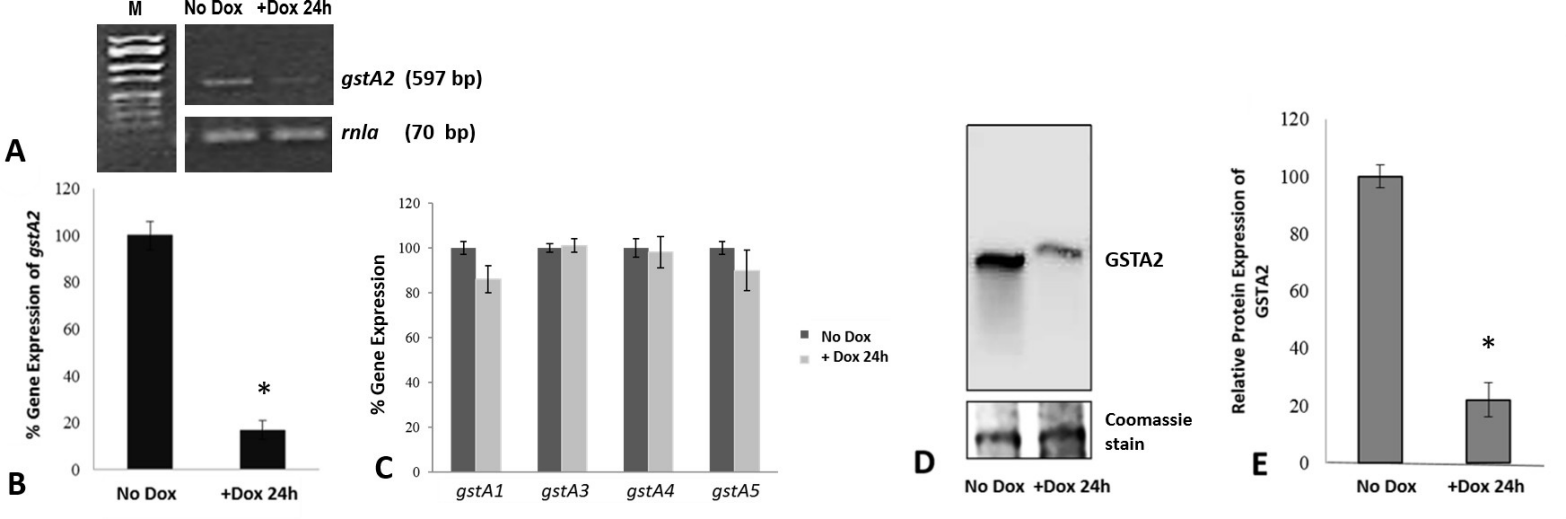
Analysis of *gstA2* expression in RNAi-*gstA2* knockdown cells. Semi-quantitative PCR analysis of 24h doxycycline-induced RNAi-specific knockdown *gstA2* gene expression showed ~82% decrease in expression of *gstA2* (A, B), other *gstAs* expression were not significantly altered (C); normalized to *rnla* internal standard. M, molecular weight markers (50–2000 bp); *rnla* (70 bp). Western blot analysis of these cells with a rabbit anti-DdGSTA2 polyclonal antibody indicated ~80% decrease in GSTA2 protein (D, E).

To explore the effect of altered GSTA2 expression on the growth and development of *D*. *discoideum*, doxycycline-inducible RNAi-*gstA2* (knock-down) and *gstA2*-OE (overexpression) AX4 strains were developed. Growth and proliferation of RNAi-*gstA2* and *gstA2-OE* AX4 in transfected and uninduced cells did not differ significantly from non-transfected AX4 cells or AX4 cells transfected with empty vector. Doxycycline induction of the RNAi-*gstA2* hairpin in vegetative log phase cells reduced *gstA2* transcripts in AX4 cells, and did not significantly affect levels of other *gstA* transcripts (Fig 7A and 7B), indicating specificity of the targeted construct to suppress the endogenous DdGSTA2 enzyme expression by as much as 80% when compared to uninduced cells (Fig 7C–7E).

**Fig 8:**
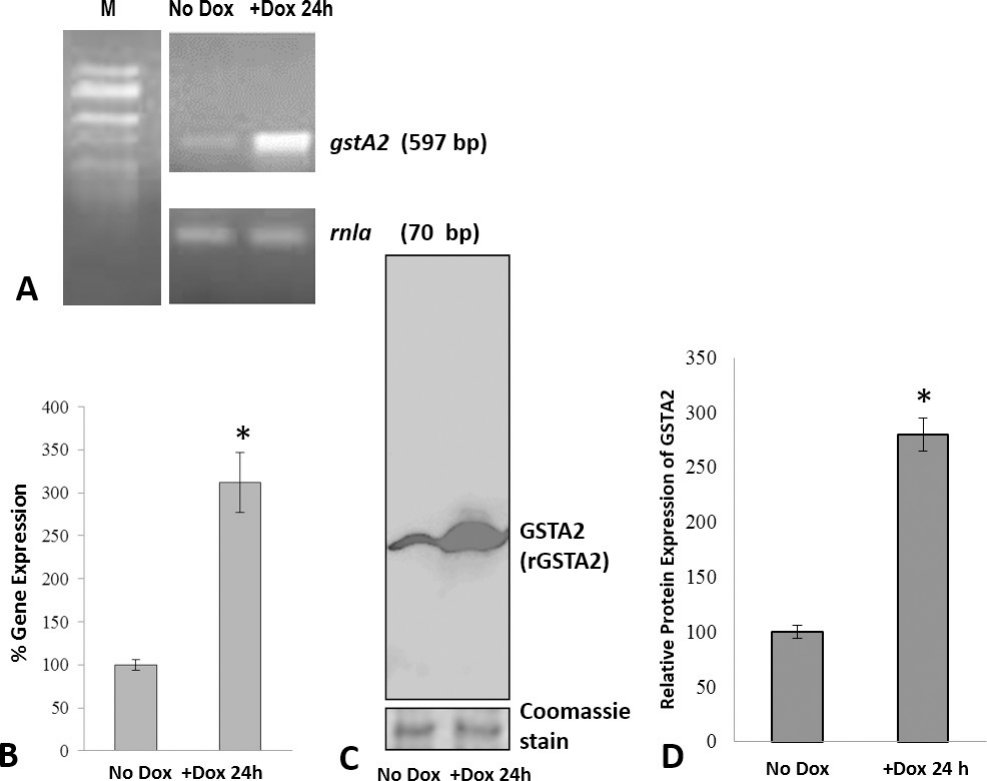
Analysis *gstA2* expression in *gstA2*-overexpression (DdGSTA2-OE) cells. (A, B) Semi-quantitative PCR analysis of doxycycline-induced expression in vegetative cells indicated a ~220% increase in the expression of *gstA2*, which was confirmed by Western blotting using rabbit polyclonal anti-DdGSTA2 (C, D). M, molecular weight markers (50–2000 bp); *rnla* (70 bp).

Doxycycline induction of the *gstA2*-OE construct in AX4 cells increased rDdGSTA2 expression by 220% and 280% (over uninduced cells) at the transcript and protein levels, respectively without doxycycline (Fig 8).

### Effects of altered DdGSTA2 levels on proliferation and development

**Fig 9:**
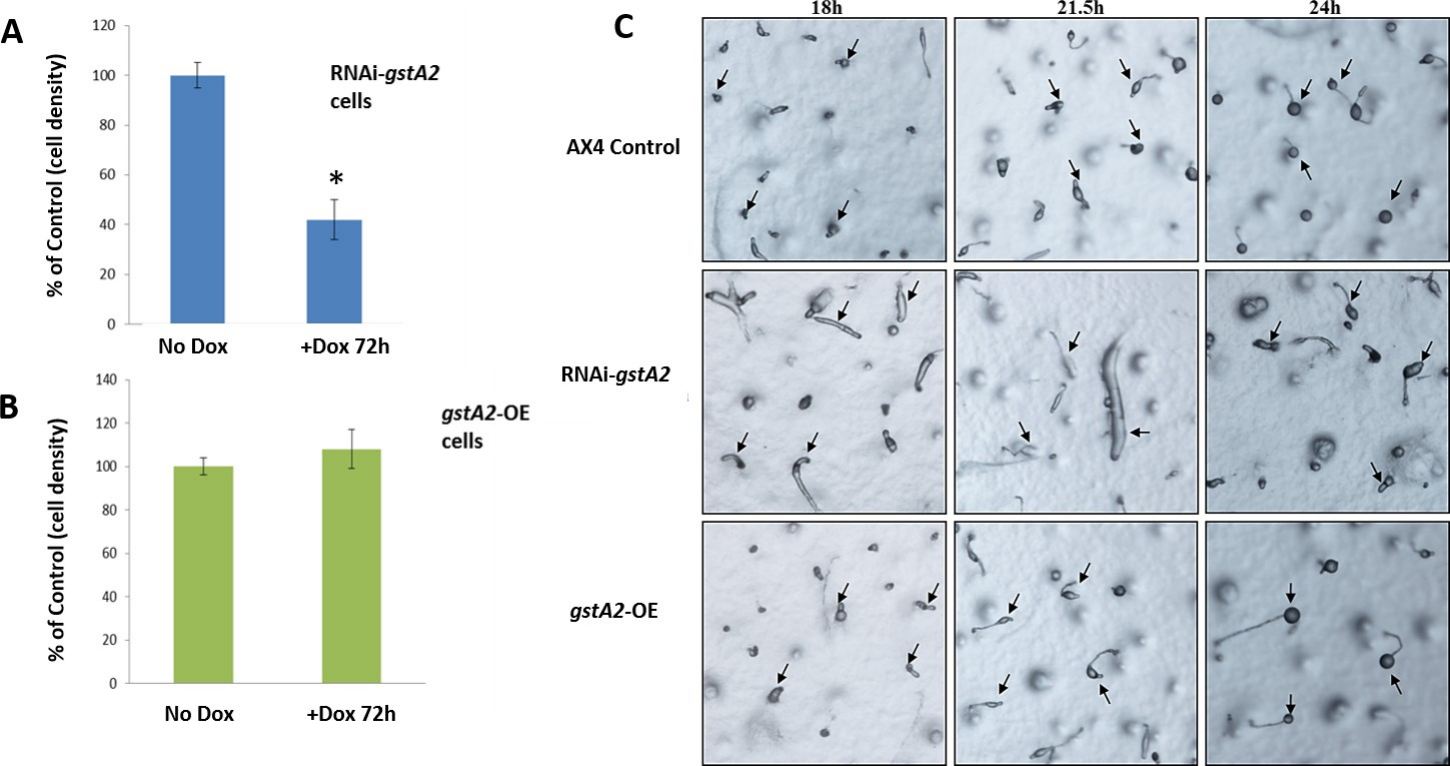
Effect of knock-down (A) and overexpression of DdGSTA2 on the proliferation and development of *D*. *discoideum*. (A) *D*. *discoideum* cell proliferation was decreased by 60% at the end of 72 hours (doxycycline-induced) *gstA2*-knockdown cells, whereas (B) overexpression of DdGSTA2 did not significantly affect the proliferation of cells. Histograms represent at least three experiments (n = 3) performed in triplicate (mean ±SD, p<0.05). (C) Developmental delays were observed in the later stages in RNAi-*gstA2* cells as compared to controls. At 18 hours the controls progressed to pre-fruiting body stage whereas RNAi-*gstA2* cells were still at slug stage. Pre-fruiting bodies observed in controls at 18 hours progressed to completion by 24 hours. RNAi-*gstA2* knockdown caused abnormalities in slug morphology (increased thickness, length, and migration) and stunted, incomplete, and fewer numbers fruiting bodies (at 24 hours). There were no developmental delays observed with DdGSTA2-overexpression when compared to controls.

RNAi-*gstA2* knockdown of the endogenous DdGST2A reduced proliferation of axenically growing cells by as much as 60% when compared to the controls after 72 hours, whereas doxycycline-induced overexpression of DdGSTA2 in *gstA2*-OE cells did not impact proliferation (Fig 9A and 9B). When starved cells were plated on non-nutrient agar in the presence or absence of doxycycline, cells expressing the RNAi-*gstA2* knockdown were similar to controls during the first 12 hours of development (aggregation and mound formation) when compared to controls, in contrast to the morphological differences observed at late stage of development/morphogenesis (predominantly at the slug stage). At 18hr of development, controls produced juvenile culminants that eventually formed fruiting bodies within 24 hours in comparison to *gstA2* (DdGSTA2) knockdown that resulted in multiple, abnormally thick slugs that retained migratory trails (Fig 9C). In general, reduced expression of DdGSTA2 caused asynchronous development, where aggregates and slugs failed to form mature fruiting bodies at 24hr. In contrast to the developmental abnormalities observed with *gstA2* knockdown/DdGSTA2 underexpression, doxycycline-induced overexpression of rDdGSTA2 did not affect stage-specific timing of development or reveal any morphological distinctions from when compared to controls. The developmental profile of each cell line is represented in Fig 9C.

## Discussion

Eukaryotic GSTs and members of their superfamily are well explored and characterized across many species. In this study we identified five *D*. *discoideum* alpha class GST transcripts (*gstA1-A5)* in unicellular, vegetative amoebae, whose expression was altered in response to starvation that induces multicellular development. Mass spectrometric analysis of the expressed GST enzymes that were isolated from axenically growing *D*. *discoideum* cells by GSH affinity chromatography revealed three of the five putative GST alpha isozymes, DdGSTA1, DdGSTA2, and DdGSTA3. The absence of DdGSTA4 and DdGSTA5 isozymes may be the consequence of reduced expression levels of each isozyme in vegetative cells, and/or stereochemical conformation(s) that inhibit their isolation with GSH binding (Fig 2A). Consistent with other members of the GST superfamily of enzymes, DdGSTs have two fundamental domains; the N-terminal domain has G-site (GSH binding site) and the C-terminal domain has H-site (hydrophobic substrate binding site) (Table 1). Tyrosine 9 is important for GSH binding and catalysis in the G-site; this amino acid is present in all GSTs of *D*. *discoideum* except for DdGSTA4, which harbors a serine residue (Table 1) [33], leading to structural folding that may render the G-site less accessible for GSH-affinity purification [34].

Expressed purified isozymes, rDdGSTA2 and rDdGSTA3, were characterized enzymatically for their kinetic parameters (K_m_ and V_max_). The V_max_ (specific activity) of rDdGSTA2 for GSH is 3.0-fold greater than rDdGSTA3. Structurally, the G-site is conserved among different organisms and the H-site, which contributes to define specificity and functions, can vary [35]. Amino acid sequence comparisons between DdGSTA2 and DdGSTA3 shows that both isozymes retain important tyrosine residues (Tyr11) within the GSH binding site, but with distinctions at Arg45/DdGSTA2 and Ile45/DdGSTA3. Additional experimentation will determine if this difference at the G-site within DdGSTA3 may be contribute to *in vivo* and *in vitro* differences in enzymatic parameters and substrate affinity between the isozymes.

Our findings demonstrate that pH 7.5 maximizes *in vitro* CDNB substrate conversion with rDdGSTA2 and rDdGSTA3 isozymes. This pH value is more alkaline than standard CDNB assay conditions (pH 6.5) used to assess GST activity across many species, and the HL5 media (pH 6.4) used for axenic growth. According to Qin and coworkers [36], the pH for maximal activity of GSTs isolated from *Locusta migratoria* ranged from 7.0 and 9.0, which may relate to the alkaline internal environment after feeding. In another study [37], cytosolic GST activity from *Corcyra cephalonica* larvae is optimum at pH 8.3. Recombinant GST from *Bombyx mori* had a broad range of optimal pH, ranging from pH 4.0 to 9.0 [38]. The soil-dwelling, natural habitat of *D*. *discoideum* makes it reasonable to hypothesize that the pH for native, cytosolic DdGSTA2 and DdGSTA3 activity may best function under slightly alkaline conditions for cultivation and survival of the organism in the wild.

The differential expression of *gstA2* at various stages of *D*. *discoideum* development indicates that DdGSTA2 regulates signaling during proliferation, chemotaxis, development, and differentiation. He and coworkers [39] reported that cell proliferation of human breast cancer cells was enhanced by plasma membrane binding of GSTπ, and increased cell viability through higher detoxification. GSTs are essential for the development of the larval stages in *Bombyx mori* [40, 41] and are important for the growth, development and shoot regeneration in plants [42]. In mammals, expression of multiclass GSTs is tissue-specific, developmentally regulated [4, 22, 23], and GST activity cooperatively promotes growth and development through antioxidant regulation. In *D*. *discoideum*, increased reactive oxygen species (ROS) during early stages of development is essential in transition from singular anatomy to multicellular development [43, 44]. Expression of the antioxidant enzymes superoxide dismutase (SOD) and catalase B, is lower in the early stages of development but increases in the later stages [44, 45], and developmental delay was observed when ROS were scavenged or with overexpression of antioxidant enzyme superoxide dismutase [44]. Garcia and coworkers [45] investigated the importance of catalase B in *D*. *discoideum* whereby *catB* null mutants had delays that were observed at later stages of development, and spores were more sensitive to hydrogen peroxide. As reported here, the transcription profile of *gstA2* is generally consistent with the expression of other *Dictyostelium* antioxidant enzymes; *gstA2* expression decreased in aggregating cells and increased in the later stages of development.

A previous study from our laboratory showed that curcumin-mediated changes in GST activity and expression correlated with changes in proliferation and development [20]. The findings herein demonstrate that knockdown of DdGSTA2 in *D*. *discoideum* alters vegetative proliferation of axenic cells and alters development of the organism. The fact that *gstA2* knockdown exerted a striking effect on slug development (18, 21.5, and 24 hours, Fig 9C) is consistent with the hypothesis that higher activity of DdGSTA2, and other antioxidant enzymes play roles across multiple stages of the *D*. *discoideum* life cycle. In contrast to the altered developmental effects observed with DdGSTA2 knockdown, the overexpression of DdGSTA2 did not affect proliferation or development. One explanation for this could be that *gstA2* transcription is already elevated vegetative cells, and additional overexpression of the isozyme may not warrant a threshold response that would result in a significant biological effect.

GST monomers conjugate with c-Jun N-terminal kinase (JNK) and control the cell proliferation and apoptotic cell death by modulating the MAPK pathway [46–49]. Overexpression of hGSTA2-2 in Leukemia cell lines (K562) decreased apoptosis by inhibiting the activation of c-jun N-terminal kinase and caspase-3 [50]. Proliferation of Caco-2 cells was decreased by overexpression of GSTA1 by modulating the JNK signaling pathway [49]. Indeed, *D*. *discoideum* homologs of MAPKs, ERK1 and ERK2 cooperatively modulate signaling pathways and regulate different stages of the life cycle [51, 52]. The functional relationship between DdGSTA2 and other isozymes to MAPKs, ERK1, and ERK2 function in *D*. *discoideum* is unknown. Recent findings that the DdGST4 isozyme functions as a DIF-1 (differentiation inducing factor-1) binding protein to regulate the sizes of cell aggregates and fruiting bodies in *D*. *discoideum* [21] provides further evidence to explore non-enzymatic roles of DdGSTs.

Amid the changes in GST expression and activity, the role of GSH should be considered, as modulation of GSH (an antioxidant) affects stage-specific development via the YakA signaling pathway, and is a substrate for GSTs [17, 18, 53]. Thus, further investigation must be conducted to determine the relationship between DdGSTA2 reduction and the YakA signaling pathway. Collectively, these observations provide a basis for further explore the function of individual DdGST enzymes in eukaryotic cell proliferation, migration, and development.

## Supporting information

S1 Raw images (TIF)

S2 Raw images (TIF)

S3 Raw images (TIF)

S4 Raw images (TIF)

S5 Raw images (TIF)
