# Supplementary figures and images for: Characterization of glutathione S-transferase enzymes in Dictyostelium discoideum suggests a functional role for the GSTA2 isozyme in cell proliferation and development
Source: PLoS One. 2021 Apr 28;16(4):e0250704. doi: 10.1371/journal.pone.0250704 (PMC8081208; doi:10.1371/journal.pone.0250704)

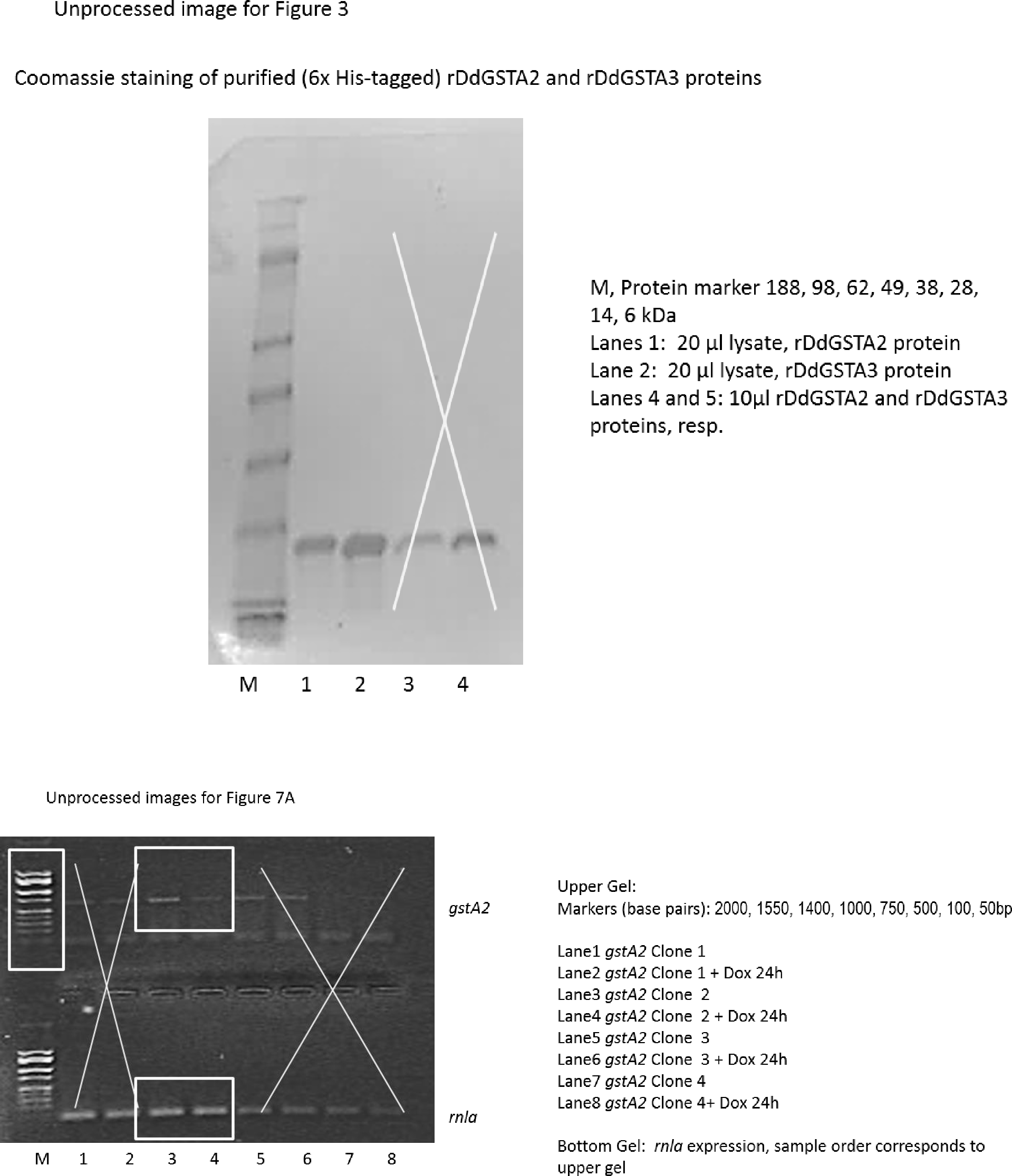

Supplement: S1 Raw images — (TIF) [file pone.0250704.s001.tif]

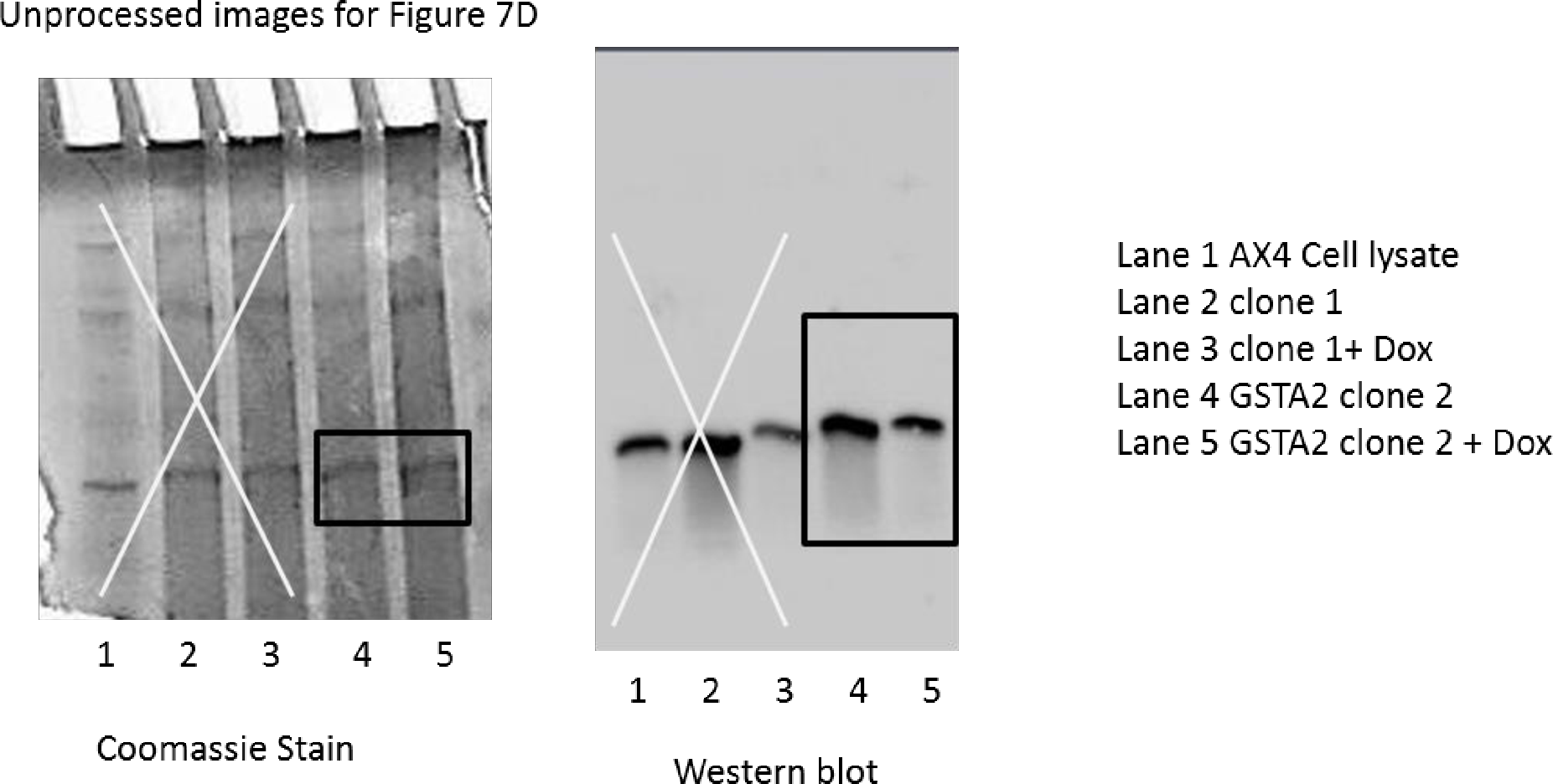

Supplement: S2 Raw images — (TIF) [file pone.0250704.s002.tif]

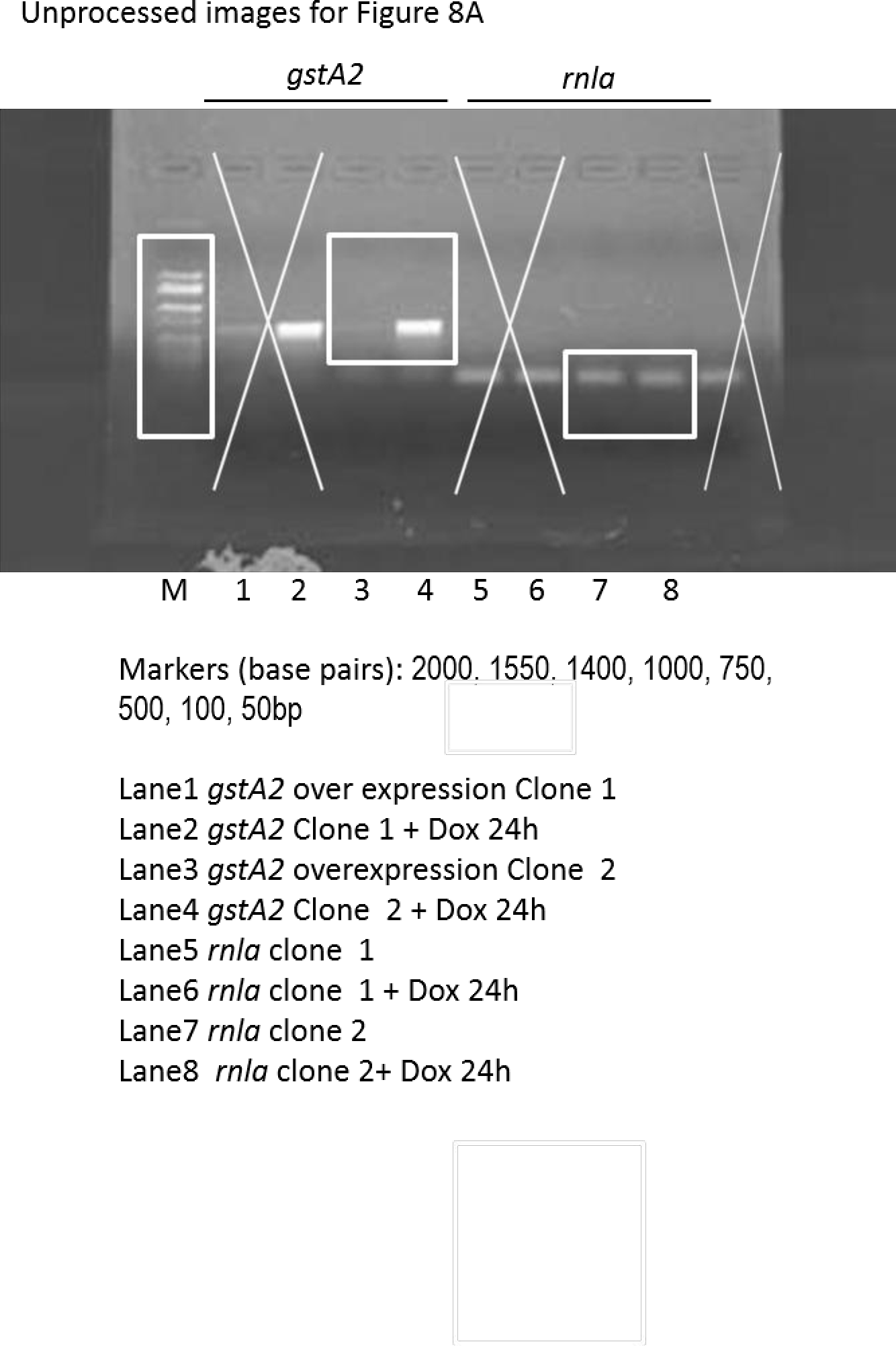

Supplement: S3 Raw images — (TIF) [file pone.0250704.s003.tif]

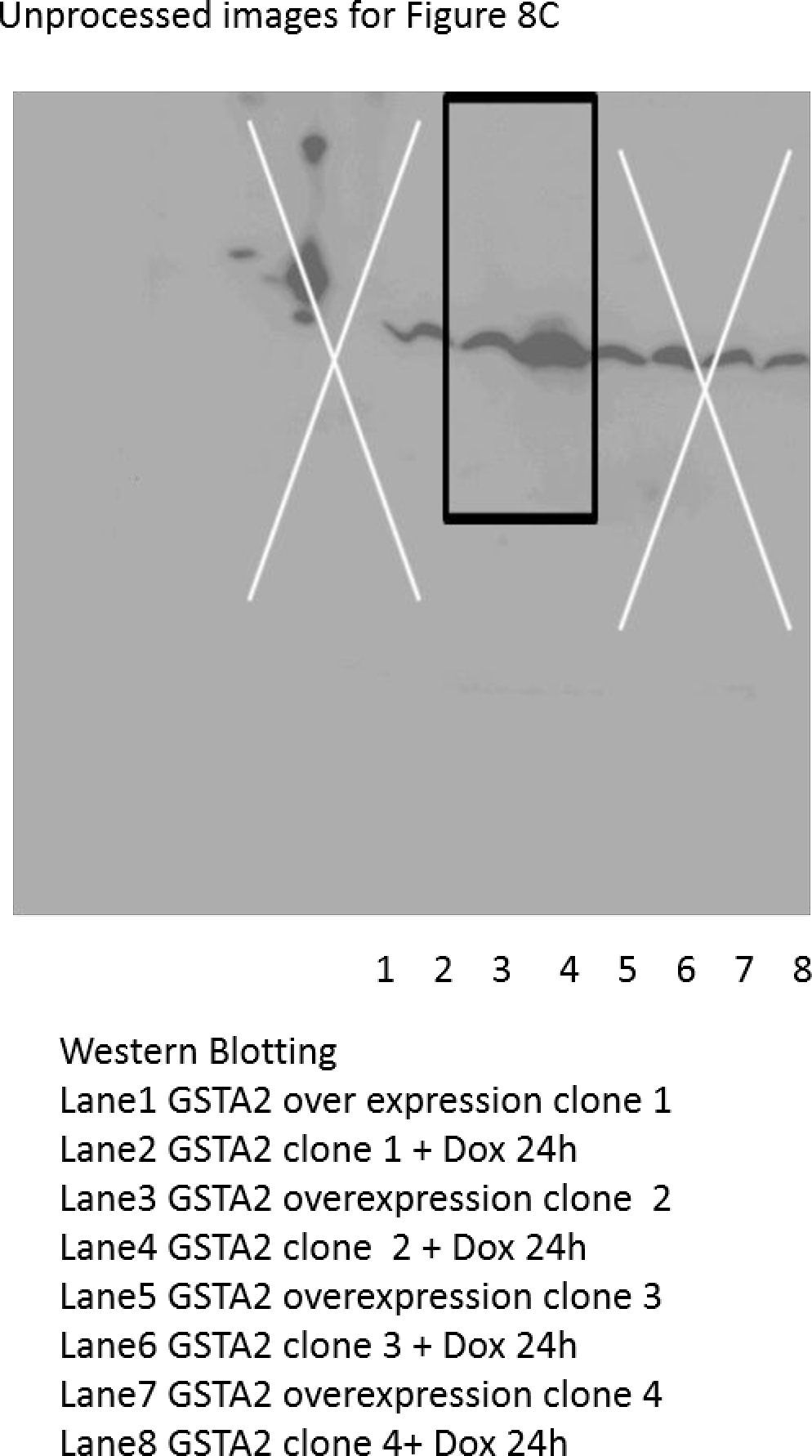

Supplement: S4 Raw images — (TIF) [file pone.0250704.s004.tif]

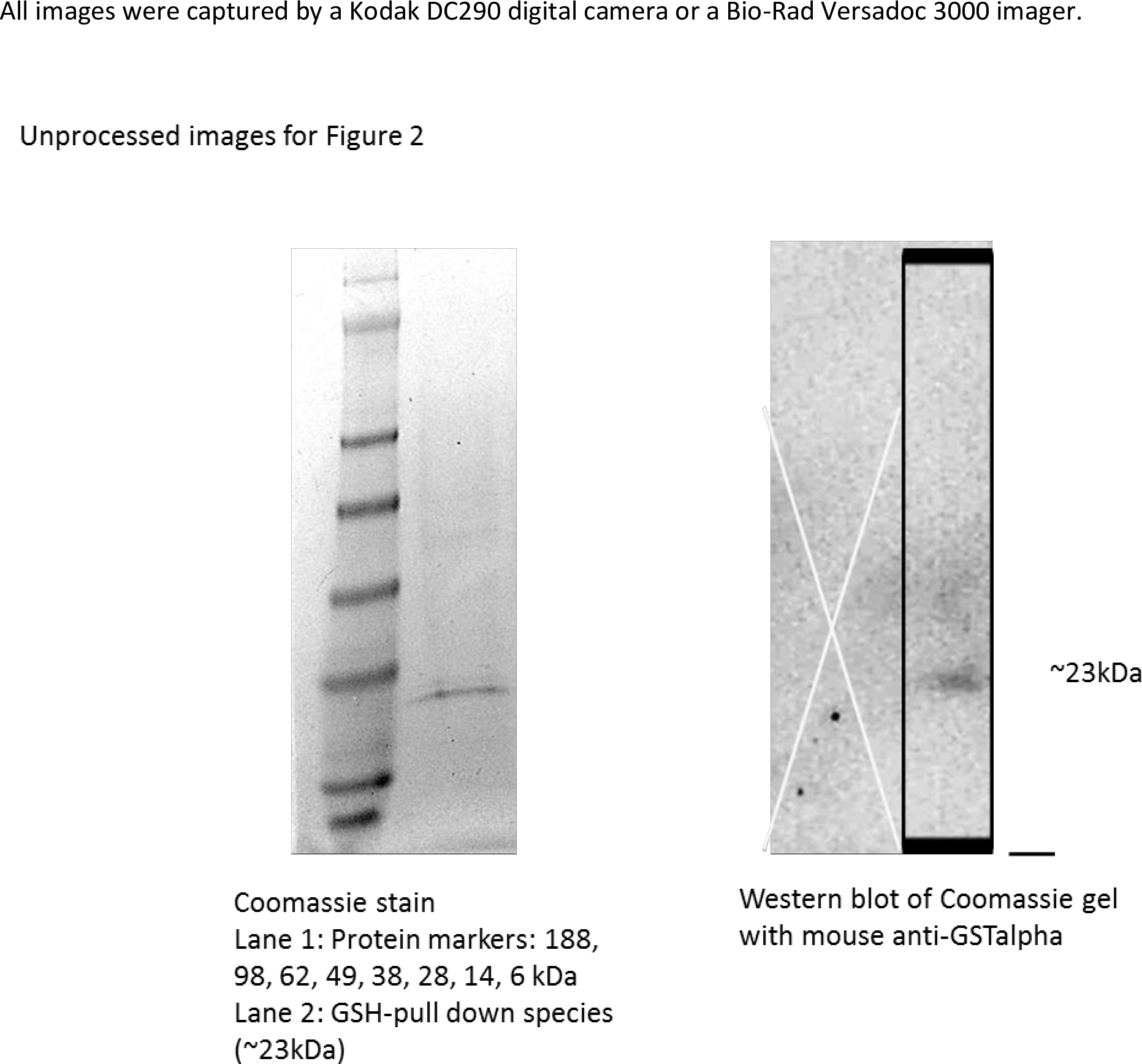

Supplement: S5 Raw images — (TIF) [file pone.0250704.s005.tif]
